# Injectable Systems for Intra-Articular Delivery of Mesenchymal Stromal Cells for Cartilage Treatment: A Systematic Review of Preclinical and Clinical Evidence

**DOI:** 10.3390/ijms19113322

**Published:** 2018-10-25

**Authors:** Alice Roffi, Norimasa Nakamura, Mikel Sanchez, Magali Cucchiarini, Giuseppe Filardo

**Affiliations:** 1 Laboratory of Nano-Biotechnology—IRCCS Istituto Ortopedico Rizzoli, 40136 Bologna, Italy; 2 Institute for Medical Science in Sports, Osaka Health Science University, Osaka 590-0496, Japan; norimasa.nakamura@ohsu.ac.jp; 3 Arthroscopic Surgery Unit-UCA, Hospital Vithas San Jose, 01008 Vitoria-Gasteiz, Spain; mikel.sanchez@ucatrauma.com; 4 Center of Experimental Orthopaedics, Saarland University Medical Center, 66421 Homburg/Saar, Germany; mmcucchiarini@hotmail.com; 5 Applied and Translational Research (ATR) Center—IRCCS Istituto Ortopedico Rizzoli, 40136 Bologna, Italy; ortho@gfilardo.com

**Keywords:** mesenchymal stromal cells, delivery vehicle, platelet-rich plasma, hyaluronic acid, hydrogels, injection

## Abstract

Stem cell-based therapy is a promising approach to treat cartilage lesions and clinical benefits have been reported in a number of studies. However, the efficacy of cell injection procedures may be impaired by cell manipulation and damage as well as by cell dissemination to non-target tissues. To overcome such issues, mesenchymal stromal cell (MSC) delivery may be performed using injectable vehicles as containment systems that further provide a favorable cell microenvironment. The aim of this systematic review was to analyze the preclinical and clinical literature on platelet-rich plasma (PRP), hyaluronic acid (HA), and hydrogels for the delivery of MSCs. The systematic literature search was performed using the PubMed and Web of science databases with the following string: “(stem cells injection) AND (platelet rich plasma OR PRP OR platelet concentrate OR biomaterials OR hyaluronic acid OR hydrogels)”: 40 studies (19 preclinical and 21 clinical) met the inclusion criteria. This review revealed an increasing interest on the use of injectable agents for MSC delivery. However, while negligible adverse events and promising clinical outcomes were generally reported, the prevalence of low quality studies hinders the possibility to demonstrate the real benefits of using such injectable systems. Specific studies must be designed to clearly demonstrate the added benefits of these systems to deliver MSCs for the treatment of cartilage lesions and osteoarthritis.

## 1. Introduction

Articular cartilage lesions occurring after traumatic injury or during progressive osteoarthritis (OA) represent a major problem in orthopedic surgery as this specialized tissue has a limited intrinsic capability for self-regeneration in absence of vascularization that may deliver reparative progenitor cells [1,2]. Thus far, none of the available clinical options, from conservative treatments to surgical approaches to restore the entire osteochondral region [3,4], have been capable of reliably and definitely allowing for the reproduction of the original hyaline cartilage in sites of damage [5,6] with a typical extracellular matrix (ECM) deposition, zonal organization, and native chondrocyte cellular phenotype that may permit functional biomechanical performance [7,8].

In this regard, stem cell-based therapy is considered to be a promising approach to treat cartilage lesions as administration of such cells in sites of injury may provide a reparative population with strong potential to commit towards the chondrocyte phenotype versus the chondrocytes themselves that tend to lose their phenotype upon expansion [9]. Mesenchymal stromal cells (MSCs) are especially good candidates to achieve this goal as they are capable of undergoing commitment events that lead to the formation of mesodermal tissues (cartilage, bone, muscle, marrow stoma, tendon, fat, dermis, connective tissues) [10,11,12] while displaying trophic, homing, and immunomodulatory activities for cells of the surrounding cartilage [13,14,15]. The following minimal set of standard criteria for characterization of MSCs have been provided by the Mesenchymal and Tissue Stem Cell Committee of the International Society for Cellular Therapy: plastic-adherence in standard culture conditions, expression of CD105, CD73, and CD90 and lack of surface expression of CD45, CD34, CD14 (CD11b), CD79α (CD19), and HLA-DR, and reliable and safe potential for differentiation mesodermal lineages (chondrocytes, osteoblasts, adipocytes) under defined culture conditions [16,17] for instance compared with embryonic stem cells (ESCs) [18] and induced pluripotent stem cells (iPSCs) [19] that carry a risk for teratoma formation and tumorigenesis [20,21]. MSCs have been isolated from a variety of tissues including the bone marrow, adipose tissue, synovium, perichondrium and periosteum, trabecular bone, umbilical cord blood, amniotic fluid, Wharton’s jelly, skeletal muscle, and even peripheral blood, all showing—at a different extent—a potential to commit towards mesodermal cells [19,22,23,24,25,26,27].

Due to the regenerative (and immunomodulatory) properties and mechanisms of action of MSCs, and considering the suboptimal results in targeting only focal lesions, a recent, increasing interest in the manipulation of MSCs in injectable approaches in order to modulate the entire joint [28]. Clinical benefits have been reported regardless of the cell source, indication, or administration method [28,29,30,31]. Still, the efficacy of the cell injection procedure may be impaired by cell manipulation and damage, as well as dissemination to non-target tissues [32,33]. To circumvent such issues, stem cell delivery may be performed using injectable vehicles as a containment system that may also provide a favorable cell microenvironment [34]. As a matter of fact, a recent study published by Desando et al. showed that the combination of MSCs with a vehicle such as hyaluronic acid (HA) modulated cell homing while promoting their attachment and integration within the damaged articular cartilage [34]. Injectable stem cell carriers that may be used for translational and clinical applications include platelet-rich plasma (PRP), platelet concentrate, HA, and a variety of hydrogel systems.

The aim of this systematic review was to analyze the available evidence on the clinical application of PRP, HA, and hydrogels for the delivery of MSCs, together with an analysis of preclinical studies that support the rationale for their use to shed light and give indications on what can be expected with such MSC-based injectable systems for the treatment of cartilage lesions and joint degeneration.

## 2. Results

**Figure 1:**
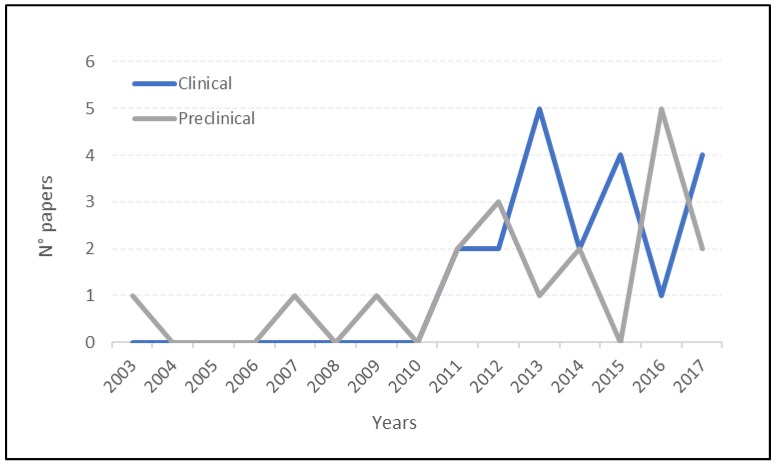
Preclinical and clinical studies published over time.

According to the search strategy, 876 papers from Web of Science and 843 papers from PubMed were screened of which 41 met the inclusion criteria (Figure 1). After full text screening, one further article was excluded since it was a clinical protocol description without any specifications on treatment results. Nineteen are preclinical papers (Table 1) and 21 clinical papers (Table 2). While the overall results of the literature search are summarized in the following paragraphs, further study details have been reported in Table 1 and Table 2 for preclinical and clinical studies, respectively.

### 2.1. Preclinical Studies

Proof-of-concept of providing regenerative MSCs via injectable carriers in vivo has been demonstrated in several preclinical animal models both of focal defects and OA. In particular, five papers reported on MSCs combined with PRP for degenerative joint disease, 12 with HA (4 for osteochondral treatment and 8 for OA), and two with hydrogels (for OA) (Table 1). Overall, these studies all supported the use of MSCs with PRP, HA, or hydrogel combination, with positive findings in both focal and degenerative joint lesions.

#### 2.1.1. MSC Injection with PRP

PRP was employed to deliver bone marrow- and fat-derived isolated or concentrated MSCs to treat degenerative joint disease in horse models, which revealed improvements in the functionality and sustainability of the damaged joints compared with PRP alone [35] as well as in the lameness score without adverse events [36]. PRP was also applied to smaller animal models of OA as a carrier of muscle- and adipose tissue-derived MSCs (also upon genetic modification), leading to improved cartilage repair versus administration of cells alone in rats [37] and rabbits [38], and to decreased lameness and inflammation with enhanced focal compressive strength relative to control treatments in dogs [39]. Overall, three [35,37,39] out of these studies directly compared the use of MSCs with or without PRP, and among them, two were able to demonstrate the benefit of using the cell carrier combination with respect to MSCs alone.

#### 2.1.2. MSC Injection with HA

HA has also been employed to inject bone marrow-derived MSCs in focal defects, promoting enhanced cartilage repair in minipigs [40], goats [41], horses [42], and rabbits [43] relative to control treatments including HA alone or cells without HA. Similarly, improved cartilage repair and reduced disease progression and inflammation were documented in animal models of OA treated with HA and bone marrow- or adipose tissue-derived MSCs in goats and sheep [44,45,46], donkeys [47], pigs [48], rabbits [34,49], and rats [50] versus HA alone or cells without HA. Overall, only four [34,43,48,50] out of these studies directly compared the use of MSCs with or without HA, and among these, three demonstrated the benefits of using the cell carrier combination with respect to MSCs alone, while one study described better results in counteracting OA progression using MSCs and HA alone compared with their combination [50].

#### 2.1.3. MSC Injection with Hydrogels

Hydrogels were also created to deliver bone marrow-derived MSCs to treat OA in rats based on self-assembling peptide hydrogels, leading to reduced inflammation and cell apoptosis as well as chondroprotection and cartilage repair [51,52] relative to control groups without cells or HA. In particular, one [52] of these studies directly compared the use of MSCs with or without hydrogel, demonstrating the benefit of using the cell carrier combination with respect to MSCs alone.

### 2.2. Clinical Studies

A number of clinical protocols were established using injectable carriers of MSCs to treat cartilage lesions and OA degeneration in patients, with a total of 21 papers meeting the inclusion criteria: 13 combined MSCs with PRP (one for osteochondral lesions and 12 for OA), four with HA (three for osteochondral lesions and one for OA), and four with a combination of PRP and HA (all for OA) (Table 2).

#### 2.2.1. MSC Injection with PRP

With regards to PRP cell delivery, three papers reported randomized clinical trials (RCT), three comparative studies, 7 case series, and one case report. Of these papers, 10 applied SVF (stromal vascular fraction) as a cell source, one cultured BMSCs, and two bone marrow aspirate concentrate (BMAC). RCT and comparative studies have been described while case series and case reports results have been summarized in the following paragraphs (details have been reported in Table 2).

As per RCT, both papers focused on OA. More in detail, in 2014 Koh et al. [62] reported on 44 patients with knee OA who underwent high tibial osteotomy followed by either PRP injection or PRP plus SVF injection, describing the best performance in pain relief and symptoms improvement in the cell group after 24 months. More recently, in 2017 Shapiro et al. [57] performed a study on 25 patients with bilateral knee OA, where the two knees were randomized into injection saline or BMAC in combination with another blood derivative (platelet-poor plasma) and followed for up to six months. Early results showed that BMAC was a safe, reliable, and viable cellular product, but study patients experienced the same relief of pain in both BMAC- and saline-treated OA knees. Similarly, in 2018 Bastos et al. [54] showed that cultured BMSCs in combination or not with PRP provided clinical improvement in patients with knee OA up to 12 months, without significant differences between groups.

The comparative studies focused on both osteochondral lesions [53] and OA [63,65]. With regard to osteochondral lesions, in 2015 Kim et al. [53] compared the outcomes of combining SVF and PRP injection versus the implantation of SVF on fibrin glue for the treatment of full-thickness lesions of the knee. Describing the improvement in both groups at final follow up (28.6 months), greater cartilage regeneration (at second-look arthroscopic, 12.4 months post-treatment) and clinical outcome improvements occurred in the implantation group rather than in those receiving a SVF injection with PRP. With regard to OA studies, in 2012 and 2013, two studies have been performed by Koh et al. [65] and Kim et al. [63] comparing surgical techniques for OA (debridement or microfracture, respectively) in combination or not with injectable therapies (SVF+PRP). Higher clinical improvements were noted at final follow-up in both studies (16.4 months and 21.8 months, respectively) for the SVF+PRP group, in particular in patients under 55 years and with an OA degree below three [65].

Case series and case reports all focused-on OA. All papers evidenced good results combining cells and PRP in terms of functional improvement and pain relief [55,56,58,59,60,61,64] together with an improvement or maintenance of cartilage appearance at second-look arthroscopy [60]. Magnetic resonance imaging (MRI) analysis showed better results with respect to the basal level in three papers [54,57,62] while Pintat et al. [56] described no complications and functional improvement but no relevant changes at MRI. Interesting, Bansal et al. [55] showed no changes in hematological and biochemical parameters before and after treatment, and a reduction of atypical cells in the synovial fluid at 24 months with respect to the basal value. Out of these studies, no one presented a control group with MSCs only, thus clear conclusions on the benefit of the carrier were not obtained.

#### 2.2.2. MSC Injection with HA

Three papers [66,67,68] reported the results of cells and HA combination on an osteochondral lesion treatment: one RCT, one comparative, and one case series where two used peripheral blood progenitor cells (PBPCs) and one used cultured bone marrow-derived MSCs. In 2013 Saw et al. [66] randomized 50 patients into two treatments: subchondral drilling and peripheral blood progenitor cells (PBPCs)+HA injections versus HA alone. PBPCs+HA resulted in better improvement of articular cartilage quality as shown by histological and MRI analyses with respect to HA alone. The non-randomized comparative study was published in 2012 by Lee et al. [67], who treated 70 patients affected by symptomatic cartilage defects with arthroscopic microfracture and intra-articular injection of cultured bone marrow-derived MSCs plus HA versus cultured bone marrow-derived MSCs and a periosteal patch implanted with an open technique. Both groups showed a similar clinical score improvement with the advantages of a minimally invasive approach for the arthroscopic technique.

The only RCT for the treatment of OA lesions was shown by Wong et al. in 2013 [69], who described the effect of cultured BMSCs in combination with HA in patients who underwent microfracture and high tibial osteotomy (HTO) with better clinical and radiographic improvement with respect to HA alone [69].

Also, the only case series [68] in this group reported good results combining subchondral drilling with PBPCs and HA injections for the treatment of chondral lesions. Out of these studies, no one presented a control group with MSCs only, thus not allowing to draw a clear conclusion on the benefit of the carrier.

#### 2.2.3. MSC Injection with PRP and HA

Finally, four papers described the results of cell/HA/PRP combination for the treatment of OA, including one RCT [70] and three case series [71,72,73]. Recently in 2017, Turajane et al. [70] described the use of autologous peripheral blood stem cells (AAPBSCs) in combination with PRP, hGCSF (human granulocyte colony-stimulating factor) and HA for the treatment of knee OA. Sixty patients were randomized into three groups: microfracture followed by injection of AAPBSCs, HA, and a mix of PRP+hGCSF or AAPBSCs, HA, and PRP versus HA injection alone. The results revealed the superiority of both AAPBSCs groups with respect to HA alone at 12 months in terms of total knee arthroplasty (TKA) avoidance and symptoms alleviation. The case series of Pak et al. [71,72,73] reported overall good clinical results for SVF plus PRP plus HA injection for the treatment of knee OA, also from the radiological point of view with cartilage-like tissue regeneration [71]. Out of these studies, no one presented a control group with MSCs only, thus clear conclusions on the benefit of the carrier were not obtained.

## 3. Discussion

This systematic research showed that MSC delivery with injectable systems (i.e., PRP, HA, hydrogels) appears to be a safe and promising treatment option with positive preclinical findings and early satisfying results in the clinical settings, although it is still not possible to draw conclusions on the entity of the added benefit of the delivery vehicle.

One of the most common solutions for an injectable system for MSCs is PRP, a blood derivative with a higher platelet concentration than whole blood. When activated, platelets release a group of biologically active proteins that bind to the transmembrane receptors of their target cells, thus leading to the expression of gene sequences that ultimately promote cellular recruitment, growth, and morphogenesis, and modulating inflammation as well [74]. This led to the wide use of PRP, showing promising results as a minimally invasive injectable treatment of cartilage degeneration and OA both in preclinical and clinical studies [75]. Among conservative treatments, viscosupplementation with HA has been also largely applied in clinical practice [3,76]. HA is a non-sulfated glycosaminoglycan (GAG) in cartilaginous ECM maintaining chondrocyte functions and a component of synovial fluid responsible for its viscoelasticy [3]. In vitro studies revealed that HA modulates inflammation inhibiting matrix metalloproteinases (MMPs) [77]. HA has potential biological effects, including the enhancement of the chondrogenic effects of MSCs [78] and the promotion of synovial cell or chondrocyte migration in the presence of basic fibroblast growth factors [79]. Finally, hydrogels derived from natural tissues are interesting recent candidates as MSC delivery agents, allowing for the invasion of cells and subsequent matrix deposition [80] with MSCs being capable of forming a cartilage specific matrix in three-dimensional hydrogel systems [80,81].

The overall positive findings explain the interest in this therapeutic strategy, with more than half of the papers published in the last four years (22/40). In particular, MSCs and PRP in combination showed positive results in preclinical settings in the animal model of both focal and degenerative joint lesions in terms of a functional improvement and decrease of inflammation and lameness, resulting in the best approach with respect to MSCs or PRP alone as supported by several in vitro studies confirming the rational of combining MSCs and PRP stimulation to induce chondrogenic differentiation with upregulation of type-II collagen genes and other articular genes (as aggrecan, Sox9, …) [82,83]. MSCs play a key role in the response to tissue injuries not only by differentiating themselves but also by inducing regenerative processes and immunomodulatory changes in the joint homeostasis through the secretion of several bioactive molecules (as anti-apoptotic, anti-inflammatory, anti-scarring/anti-fibrosis molecules) [84]. Similarly, PRP can influence all joint tissues, offering the possibility to deliver a high concentration of autologous growth factors and bioactive molecules in physiologic proportions that are critical to regulate tissue healing processes and modulate inflammation [75]. Thus, the possibility to use these products in combination for an injection approach may further enhance the healing processes with respect to both biological products. Overall positive results were obtained upon intra-articular delivery of MSCs/PRP and in all papers their combined use can be consider safe as no major adverse events related to the treatment were reported. Bansal et al. [55] performed a synovial fluid analysis from patients undergoing PRP+MSC treatment, showing a reduction of atypical cells after treatment and no abnormalities in hematological and biochemical parameters before and after 24 months of treatment.

Similarly, in MSCs and HA combination, preclinical results are overall positive in both animal models of focal and degenerative lesions, in particular in terms of the reduction of inflammation and disease progression [34,44,45,46,47,48,49]. In the clinical settings, all papers described the treatment of osteochondral lesions, showing safety and positive results and the regeneration of hyaline-like cartilage over the same protocol without cells [66]. A study by Lee et al. [67] described similar results between the surgical and injection approach, with the advantage of intra-articular injection being minimally invasive. For the treatment of degenerative lesions, only RCT [69] reported clinical and radiographic improvement in cells group with respect to HA alone, although the lack of cells in the alone group prevented useful information on the MSC carrier from being obtained.

The use of hydrogels as agents for MSC delivery was only described in the preclinical setting for OA treatment, showing good results in terms of improved cartilage regeneration and decrease in inflammation scores. Hydrogels are gaining attention as ECM mimicry systems due to their water content and water-swollen networks that facilitate the transport of water-soluble biomolecules [85]. Such a treatment approach is still in early development and not yet translatable into a clinical protocol, as there is no current material capable of addressing all the mechanical challenges of injection approach [85], and some further aspects need to be clarified including proper hydrogel viscosity, cells concentration, and application protocol.

Another interesting approach applied only in the clinical setting is the combination of both PRP and HA for MSC delivery. The use of HA in combination with PRP for OA treatment is gaining increasing attention in the scientific community, both in the preclinical and clinical settings, reporting overall good results in terms of anti-inflammatory effects [86,87] and pain and function improvement [88]. The rationale to combine these treatments systems as a carrier is related to their dissimilar biological mechanisms and to the rheological properties of HA which, together with the regenerative potential of PRP and MSCs, which might lead to a more effective treatment for OA [89].

Finally, this systematic review also underlined another interesting finding. While in the preclinical papers expanded cells from different sources have been commonly used, in the clinical papers the main cell type and source used is the minimum manipulated SVF from the adipose tissue, probably due to the ease of collection and to economical, ethical, and regulatory issues [28]. To date, there is no clear evidence about the most suited source of MSCs for joint tissues regeneration, the best procedure to obtain them, and the optimal cells dosage. The heterogeneous results presented in this systematic review actually confirm the confounding background and it is difficult to draw a clear conclusion on the best processing methods, MSC type and dosage, and their effect on joint tissues regeneration. Moreover, another unclear aspect is the composition of products for intra-articular injection in terms of cell number and type, when the MSCs are used as a concentrate [28]. Regardless of the products injected, overall promising results were noted when combining MSCs with agents for joint delivery, but the real potential of these options needs to be confirmed by high quality comparative clinical studies with wider patient population, extended periods of evaluation, investigating also the proper cell dosage and source to be delivered. In fact, while there is some preclinical evidence with comparative studies showing the importance of delivery systems, the clinical literature is lacking specific treatment groups to identify their contributions to the outcomes, which makes their clinical usefulness questionable considering that overall good results have also been reported in clinical studies upon injection of MSCs without vehicles [28]. Further comparative studies will thus be needed in the future to confirm the role of injectable systems and to optimize their function in clinical practice. Finally, the effect of such vehicles will have to be compared with the new frontiers of MSC delivery: in fact, current research is exploring the potential to improve MSC treatment by retaining them in target tissues long enough to induce the regeneration process through modern techniques of MSC magnetic labelling with an external device to permit cell localization in a specific area as reported by Kamei et al. [90].

This systematic review of the literature revealed several limitations, such as the heterogeneity of both preclinical and clinical papers characterized by different delivery approaches, anatomical locations, animal model, and small patient population, which makes it difficult to draw any final conclusion about the best treatment strategy for the treatment of focal and degenerative cartilage lesions. Moreover, another key weakness is the study designs, which mainly focused on the combined use while rarely comparing groups to demonstrate the benefit of adding an MSC vehicle for injections.

Specific studies should be designed to demonstrate the improvement provided by injectable systems to deliver MSCs, as well as to identify the optimal source of cells (both in terms of ease of collection and cost-effectiveness, as well as differentiation potential and resistance-responsiveness to the inflammatory OA environment), their manipulation, dosage, the indications on pathology, and the disease phase with the aim of optimizing the outcome for a treatment of focal osteochondral lesions or joint degeneration.

## 4. Materials and Methods

**Figure 2:**
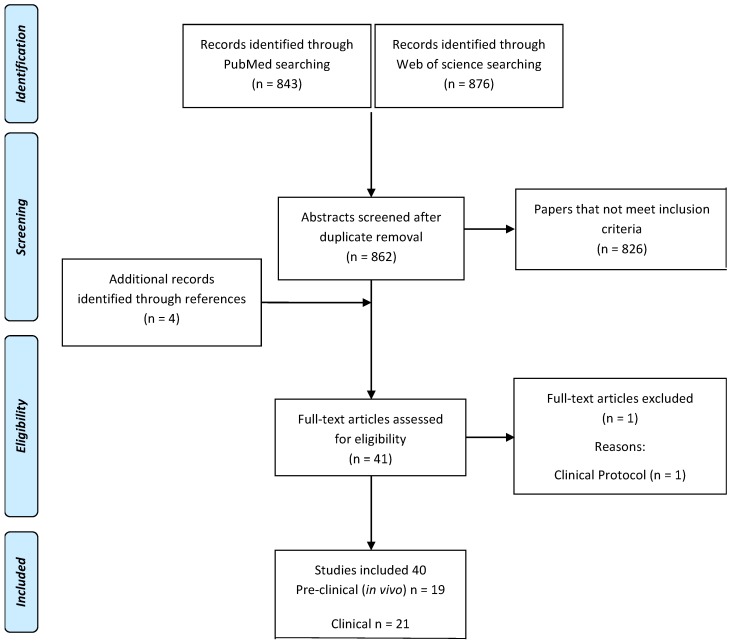
Flowchart of literature search.

The systematic literature search was performed using the PubMed and Web of Science databases with the following string: “(stem cells injection) AND (platelet rich plasma OR PRP OR platelet concentrate OR biomaterials OR hyaluronic acid OR hydrogels)”, selecting papers in the English language published up to August of 2018 on the use of MSCs in combination with PRP, HA, or hydrogel injectable vehicles for joint delivery in the preclinical (in vivo) and clinical field for the treatment of cartilage pathology (focal defects or OA). Articles were first screened by title and abstract by two independent observers, subsequently, the full texts of the resulting articles were analyzed. Reference lists from the selected papers were also screened to identify further articles (Figure 2).

## 5. Conclusions

This systematic review revealed an increasing interest on the use of injectable agents for MSC delivery. While negligible adverse events and promising clinical outcomes were generally reported, the prevalence of low quality studies hinders the possibility to demonstrate the real benefits provided by combining MSCs with injectable systems. Many variables also need to be envisaged, such as most effective delivery agents, cell source, dosage, and most suitable application protocol. Finally, it will be important to identify which patients may benefit more from these procedures, before a wide application of this promising biological approach can occur in clinical practice for the treatment of cartilage lesions and OA degeneration.

## Figures and Tables

**Table 1:** Details of preclinical papers identified in the systematic research.

Publication	Animal Model	Lesion Type	MSC Type	Delivery System	Study Design	Results
**PRP**						
Bembo 2016 [36] Muscles, ligaments and tendon J	Sport horses	OA	Micro fat suspension	PRP Plts 4.3 ± 1.1 × 10^9^/L Activation: No Leucocytes: 20 ± 9 × 10^6^/L	Micro fat+PRP Experimental time: 3 months	Significant improvement of the lameness score 3 months after treatment; returned to competition for 4 horses which 3 resumed intensive training; no adverse events
Broeckx 2014 [35] PLoS ONE	Sport horses		Allogeneic PBMSCs	PRP Plts 200 × 10^6^; stored at −80 °C before use Activation: N/A Leukocytes: N/A	PRP vs. MSCs vs. MSCs+PRP vs. chondrogenic induced MSCs+PRP Experimental time: 6 and 12 weeks, 6 and 12 months	Significant function improvement up to 12 months after treatment in MSCs+PRP group compared with PRP alone. Highest short-term clinical scores were obtained with chondrogenic induced MSCs+PRP
Hermeto 2016 [38] Genet Mol Res	Rabbits		AD-MSCs (4 × 10^6^ cells)	PRP Plt: 997.42 ± 48.01/μL; Activation: 10% Ca gluconate Leukocytes: N/A	Saline vs. PRP vs. undifferentiated MSCs+PRP vs. differentiated MSCs+PRP Experimental time: 2 months	Improved tissue repair in both MSCs group at macroscopic and histological examinations; any improvements in PRP alone group
Yun 2016 [39] J Orthop Surg Res	Dogs		AD-MSCs (10^7^ cells)	PRP Plts: 10^6^/μL Activation: N/A Leukocytes: N/A	Saline vs. PRP vs. MSCs+saline vs. MSCs+PRP Experimental time: 2, 3, 4 months	Decreased lameness score at 2 and at 3 months in both PRP alone and MSCs+PRP groups; significant increases in focal compressive strength in all treatments groups with highest value in MSCs+PRP group; inflammation reduction in both PRP and MSCs+PRP groups
Mifune 2013 [37] Osteoarthritis Cartilage	Rats		MDSCs (5 × 10^5^ cells)	PRP Plts: 230 × 10^4^/mL) Activation: N/A Leukocytes: N/A	Saline vs. PRP vs. MDSCs vs. MDSCs expressing BMP-4/sFlt1+PRP vs. MDSCs expressing BMP-4/sFlt1 vs. MDSCs+PRP Experimental time: 4 and 12 weeks	Significant AC repair at histology in MDSCs expressing BMP-4/sFlt1+ PRP at 4 weeks compared with MDSCs expressing BMP-4/sFlt1, with higher numbers of cells producing type-II collagen and lower levels of chondrocyte apoptosis
**HA**						
Kim 2012 [43] Knee Surg Relat Res	Rabbits	Osteochondral defect	BMSCs (10^6^ cells)	HA	No treatment vs. HA vs. MSCs vs. MSCs+HA vs. MSCs+HA inj vs. MSCs+HA+1 HA inj vs. MSCs+HA+2 HA inj Experimental time: 7 weeks	Significant improvements in osteochondral defect healing at macroscopic and histological evaluation in all treatment groups compared with untreated defects; at histology, MSCs+HA+2 HA inj showed better results than other groups
McIlwraith 2011 [42] Arthroscopy	Horses		BMSCs (20 × 10^6^ cells)	High molecular weight HA	MFX+HA or MFX+HA+MSCs Experimental time: 6 and 12 months	No difference in clinical and histological analysis, but significant increase in repair tissue firmness and better repair tissue quality at arthroscopic and macroscopic analysis in MSCs group with greater levels of aggrecan than in HA alone group
Saw 2009 [41] Arthroscopy	Goats		BMC	High molecular weight HA	No treatment vs. subchondral drilling + 3 HA inj vs. subchondral drilling + 3 HA + BMC inj Experimental time: 24 weeks	Better cartilage repair in MSCs group at histology, with hyaline cartilage regeneration
Lee 2007 [40] Stem Cells	Minipigs		BMSCs (3.5–10.1 × 10^6^ cells)	High molecular weight HA	Saline vs. HA vs. MSCs+HA Experimental time: 6 and 12 weeks	Improvement in cartilage healing at histologic and macroscopic analysis at both 6 and 12 weeks in MSCs+HA group compared with controls
Lv 2018 [46] Cell Transplant	Sheep	OA	SVF vs. cultured AD-MSCs	Medium molecular weight HA	Saline vs. HA vs. SVF/HA vs. low dose AD-MSCs/HA vs. high dose AD-MSCs/HA Experimental time:	Better results in AD-MSCs/HA than SVF/HA in blocking OA progression and promoting cartilage regeneration
Feng 2017 [45] Tissue Eng Part A	Sheep		Allogeneic AD-MSCs (5 × 10^7^ cells vs. 10^7^ cells)	Medium molecular weight HA	High dosage AD-MSCs or low dosage + HA vs. HA alone vs. saline Experimental time: 14 weeks	Typical articular cartilage feature in both AD-MSCs groups and presence of AD-MSCs at synovium at 14 weeks at MRI; lower inflammatory factors from synovial fluid of AD-MSCs groups than HA alone
Desando 2017 [34] Tissue Eng Part C	Rabbits		BMSCs (2 × 10^6^ cells) and BMC	High molecular weight HA	BMSCs+saline vs. BMSCs+HA vs. BMC+saline vs. BMC+HA Experimental time: 2 months	Joint repair evidence in all treatments, superior results for BMC-HA than other groups; BMSCs migrate to the meniscus while BMC in cartilage, but HA favor cells migration to cartilage
Chiang 2016 [49] Plos ONE	Rabbits		Allogeneic BMSCs (10^6^ cells)	High molecular weight HA	Untreated vs. Sham vs. HA vs. MSCs+HA Experimental time: 6 and 12 weeks	Less cartilage loss and surface abrasion with better histological scores and cartilage content in MSCs group compared with HA alone; engraftment of allogenic MSCs were evident in surface cartilage
Suhaeb 2012 [50] Indian J Exp Biol	Rat		BMSCs (3-5 × 10^6^ cells)	High molecular weight HA	HA vs. BMSCs vs. BMSCs+HA	Better results with HA and BMSCs alone in counteracting OA progression with respect to their combination
Sato 2012 [48] Arthritis Res Ther	Pigs		Xenogeneic hMSCs (7 × 10^6^ cells)	Low molecular weight HA	Saline vs. HA vs. MSCs+saline vs. MSCs+HA Experimental time: 5 weeks	Histological partial defect repair only in MSCs+HA group at 5 weeks with an increase in type-II collagen content and low levels of MMP-13
Mokbel 2011 [47] BMC Musculoskeletal Disorders	Donkeys		BMSCs (1.8-2.3 × 10^6^ cells/mL)	Low molecular weight HA	MSCs+HA vs. HA alone Experimental time: 1, 2, 6 months	Defect repair at clinical and radiological evaluation in MSCs+HA group compared with the control; MSCs integrated with healthy cartilage in the superficial and inner part
Murphy 2003 [44] Arthritis Rheum	Goats		BMSCs (10 × 10^6^ cells)	High molecular weight HA	HA vs. HA+BMSCs Experimental time: 12 and 26 weeks	No adverse events; stimulation of the regeneration of meniscal tissue and delay of OA progression in MSCs group
**Hydrogel**						
Kim 2016 [51] Biomaterials	Rat OA	OA	PBMSCs	SAP hydrogel	SAP hydrogel 0.5 SP vs. SAP hydrogel SP vs. SAP hydrogel 2SP vs. SAP hydrogel SP+MSCs Experimental time: 6 weeks	Markedly improved cartilage regeneration in the SAP-SP group showing recruitment of MSCs in the defect
Kim 2014 [52] Int J Nanomed	Rat OA		Allogeneic BMSCs	SAP hydrogel	MSCs vs. SAP hydrogel vs. SAP hydrogel+MSCs vs. no treatment Experimental time: 6 weeks	Evidence of chondroprotection at histological view and decrease of inflammation and apoptosis biomarkers in SAP+MSCs group; increased BMD in SAP hydrogel+MSCs groups relative to the controls

**Abbreviations:** adipose-derived mesenchymal stromal cells, AD-MSCs; articular cartilage, AC; hyaluronic acid, HA; magnetic resonance imaging, MRI; platelet rich plasma, PRP; degenerative joint disease, DJD; peripheral blood MSCs, PBMSCs; self-assembled peptide, SAP; substance P, SP; bone marrow concentrate, BMC; bone marrow-derived MSCs, BMSCs (cultured); matrix metalloproteinase, MMP; muscle-derived MSCs, MDSCs; human umbilical cord blood MSCs, hUCB-MSCs; extracellular matrix, ECM; microfracture MFX; bone mineral density, BMD; stromal vascular fraction, SVF.

**Table 2:** Details of clinical papers identified in the systematic research.

Defect type	Publication	Study Type	MSC Type	Delivery System	Study Design	Results
**PRP**						
Osteochondral lesion	Kim 2015 [53] Am J Sports Med	Comparative	SVF	PRP Plts: 1.28 × 10^6^/μL Activation: CaCl_2_ Leukocytes: N/A	40 pts (20 vs. 20) (knee) Age: mean 59.2 years Lesion size/degree: 5.44 ± 1.4 cm^2^ Treatment: SVF on FG scaffold vs. SVF+PRP Follow-up: 28.6 months	Significant improvement in both groups; better clinical results at final follow-up and 2nd look appearance at 12 months for SVF-FG
OA	Bastos 2018 [54] KSSTA	RCT	Cultured BMSCs	PRP Plts: 10^6^/μL Activation: N/A Leukocytes: N/A	18 pts (9 vs. 9) (knee) Age: mean 57.6 years Lesion size/degree: grade II-IV Treatment: BMSCs vs. BMSCs+PRP Follow-up: 12 months	Improvement in knee pain and function in both groups, without significance difference
	Bansal 2017 [55] J Transl Med	Case series	SVF	PRP Plts: N/A Activation: N/A Leukocytes: N/A	10 pts (knee) Age: mean 58.4 years Lesion size/degree: N/A Treatment: SVF+PRP Follow-up: 3, 6, 12, 18, 24 months	Functional improvement with pain reduction at 12 and 24 months; reduction of atypical cells in synovial fluid; unaltered haematological and biochemical analysis
	Pintat 2017 [56] J Vasc Interv Radiol	Case series	SVF	PRP Plts: 700,000/mm^3^ Activation: N/A Leukocytes: 200/mm^3^	19 pts (knee) Age: mean 42.1 years Lesion size/degree: N/A Treatment: SVF+PRP Follow-up: 6 and 12 months	Functional improvement at 6 and 12 month follow-ups with no complications but no relevant changes at MRI
	Shapiro 2017 [57] Am J Sports Med	RCT	BMAC	PPP	25 pts (knee) Age: mean 60 years Lesion size/degree: 2.3 K-L Treatment: BMC+PPP vs. saline Follow-up: 6 months	No adverse events; similar pain relief in both group
	Gibbs 2015 [58] J Pain Res	Case series	SVF	PRP Plts: N/A Activation: N/A Leucocytes: N/A	4 pts (7 knees) Age: mean 51.5 years Lesion size/degree: N/A Treatment: SVF+PRP and 3 monthly PRP inj Follow-up: 12 months	Functional, pain and quality of life score improvement at 12 months
	Srinivas 2015 [59] J of evolution of med and Dent Sci	Case series	BMC	PRP Plts: N/A Activation: N/A Leucocytes: N/A	115 pts (knee) Age: 56–87 years Lesion size/degree: moderate to severe Treatment: 65 BMC+PRP and 50 corticosteroid Follow-up: 6 months	Pain improvement from 1 week up to 6 months after injection of PRP + BMC
	Koh 2015 [60] KSSTA	Case series	SVF	PRP Plts: 1.28 × 10^6^ cells/μL Activation: N/A Leucocytes: N/A	30 pts (knee) Age: mean 70.3 years Lesion size/degree: 2.3 K-L Treatment: SVF+PRP Follow-up: 24 months	Significant clinical improvement; 87.5% of 2nd look arthroscopy within 24 months improved or maintained cartilage status
	Pham 2014 [61] Biomed Res Ther	Case series	SVF	PRP Plts: N/A Activation: CaCl_2_ Leukocytes: N/A	21 pts (knee) Age: N/A Lesion size/degree: II/III Treatment: SVF+PRP Follow-up: 6 months	Significant clinical scores improvement; no side effects; increased cartilage thickness at MRI
	Koh 2014 [62] Arthroscopy	RCT	SVF	PRP Plts: 1.303 × 10^3^ mL Activation: N/A Leukocytes: N/A	44 pts (21 vs. 23) (knee) Age: mean 53.2 years Lesion size/degree: 1–3 K–L Treatment: HTO+PRP vs. HTO+PRP+SVF Follow-up: 24 months	Better improvement of KOOS pain and symptoms and VAS pain in SVF+PRP than PRP alone
	Kim 2013 [63] Am J Sports Med	Comparative	SVF	PRP Plts: N/A Activation: N/A Leukocytes: N/A	75 pts (ankle) Age: mean 56.8 years Lesion size/degree: 108.76 ± 34.6 mm^2^ Treatment: MFX vs. MFX+SVF+PRP Follow-up: 21.8 months	Clinical improvement in both groups with better results for SVF group
	Koh 2013 [64] Arthroscopy	Case series	SVF	PRP Plts: 1.28 × 10^6^/μL Activation: CaCl_2_ Leukocytes: N/A	18 pts (knee) Age: mean 54.6 Lesion size/degree: ICRS grade 3 or 4 Treatment: SVF+PRP Follow-up: 24.3 months	Function and pain improvement WOMAC and MRI correlate with cell numbers, better if OA < 3
	Koh 2012 [65] Knee	Comparative	SVF	PRP Plts: 1.28 × 10^6^/μL Activation: CaCl_2_ Leukocytes: N/A	50 pts (knee) Age mean: N/A Lesion size/degree: ICRS grade mean 3.2 Treatment: debridement vs. debridement+SVF+PRP+2 weekly PRP inj Follow-up: 16.4 months	No major adverse events; improvement of clinical scores in both groups; SVF performed better at < 55 years and OA < 3
**HA**						
Chondral lesion	Saw 2013 [66] Arthroscopy	RCT	PBPCs	High molecular weight HA	50 pts (25 vs. 25) (knee) Age: mean 40 years Lesions size: ICRS grade 3 and 4 lesions Treatment: Subchondral drilling + 5 weekly inj of PBPCs+HA vs. HA alone+3 weekly inj after 6 months Follow-up: from 18 to 24 months	Improvement of the quality of articular cartilage repair in PBSC group at histologic and MRI evaluation
	Lee 2012 [67] Ann Accad Med	Comparative	Cultured BMSCs	High molecular weight HA	70 (35 vs. 35) (knee) Age: mean 44 Lesion size: N/A Treatment: MFX+inj of BMSCs+HA (+ 2 weekly inj HA) vs. BMSCs+periosteal patch Follow-up: 24.5 months	No significant difference between the two procedures, with less invasivity for BMSCs/HA IA inj
	Saw 2011 [68] Arthroscopy	Case series	PBPCs	High molecular weight HA	5 pts (knee) Age: mean 39.4 years Lesions size: 2 grade IV kissing lesions–3 small Treatment: Subchondral drilling+PBPCs+HA 5 weekly inj Follow-up: from 10 to 26 months	No adverse events; hyaline cartilage regeneration at histology
OA	Wong 2013 [69] Arthroscopy	RCT	Cultured BMSCs	N/A	56 pts (28 vs. 28) (knee) Age mean: mean 51 years Lesion size/degree: Treatment: BMSCs+HA vs. HA inj after MFX+HTO Follow-up: 24 months	Clinical improvement at short term and MOCART outcomes at 1 year in cells group
**Combination of delivery agents**						
OA	Turajane 2017 [70] Stem Cells Int	RCT	AAPBSCs	PRP Plts: N/A Activation: N/A Leukocytes: N/A High molecular weight HA	60 pts (20 vs. 20 vs. 20) Age: mean 56.5 years Lesion size/degree: 2.3 K-L Treatment: MFX + 3 weekly inj of AAPBSCs+HA+PRP+hGCSF vs. MFX+3 weekly inj of AAPBSCs+HA+PRP vs. 3 weekly inj HA alone Follow-up: 12 months	Avoidance of TKA in the AAPBSC groups at 12 months and potent, early, and sustained symptom alleviation in GFA groups vs. HA alone
	Pak 2016 [71] BioRes Open Access	Case series	SVF	PRP Plts: N/A Activation: CaCl_2_ Leukocytes: N/A	3 pts (knee) Age: mean 71.6 years Lesion size/degree: stage 3 OA Treatment: SVF+PRP+HA+3 weekly PRP inj Follow-up: 3 months	Function and pain improvement at 3 months with signs of regenerating cartilage-like tissue at MRI
	Pak 2013 [72] BMC Musculoskeletal Disord	Case series	SVF	PRP Plts: N/A Activation: CaCl_2_ Leukocytes: N/A	91 pts (various anatomic locations) Age: mean 51.23 years Lesion size/degree: N/A Treatment: SVF+PRP+HA+4 weekly PRP inj Follow-up: 26.62 months	SVF/PRP injections are safe; clinical improvement of knee and hip
	Pak 2011 [73] J Med Case Rep	Case report	SVF	PRP Plts: N/A Activation: CaCl_2_ Leukocytes: N/A	2 pts (knee), 2 pts (hip) Age: 70, 79, and 29, 47 years Lesion size/degree: N/A Treatment: SVF+PRP+low dose dexamethasone inj Follow-up: 3 months	Clinical improvement; significant positive changes at MRI

**Abbreviations:** peripheral blood progenitor cells, PBPCs; hyaluronic acid, HA; randomized controlled trial, RCT; not available, N/A; fibrin glue, FG; bone marrow aspirate concentrate, BMAC; high tibial osteotomy, HTO; microfracture, MFX; bone marrow concentrate, BMC; platelet poor plasma, PPP; autologous activated peripheral blood stem cells, AAPBSCs; bone marrow-derived mesenchymal stromal cells, BMSCs; stromal vascular fraction, SVF.
